# The long noncoding RNA LINC00483 promotes lung adenocarcinoma progression by sponging miR-204-3p

**DOI:** 10.1186/s11658-019-0192-7

**Published:** 2019-12-16

**Authors:** Shengzhuang Yang, Tao Liu, Yu Sun, Xiangsen Liang

**Affiliations:** Department of Chest Cardiovascular Surgery, The Second Affiliated Hospital of Guangxi Medical University, 166 University East Road, Xixiangtang District, Nanning City, 530007 Guangxi Province China

**Keywords:** LINC00483, Lung adenocarcinoma, Cell proliferation, Cell migration/invasion, ETS1

## Abstract

**Background:**

The expression of the long noncoding RNA LINC00483 is upregulated in lung adenocarcinoma (LUAD). However, its role in the progression of LUAD and the underlying mechanisms remain elusive.

**Methods:**

The expressions of LINC00483 and miR-204-3p were determined using quantitative real-time PCR. The correlation between the clinicopathological characteristics of LUAD patients and LINC00483 expression was analyzed using Pearson’s χ^2^ test. A549 and PC-9 cells were transfected with small interfering RNA (siRNA) that specially targeting LINC00483 to assess the impact of its knockdown. Cell proliferation was assessed using the Cell Counting Kit-8 and clone forming assays. Cell migration and cell invasion were evaluated using a transwell assay. The levels of Snail, E-cadherin, N-cadherin and ETS1 proteins were determined via western blotting. The interaction between LINC00483 and miR-204-3p was analyzed using dual-luciferase, fluorescence in situ hybridization and RNA immunoprecipitation.

**Results:**

LINC00483 was upregulated in LUAD tissues and cell lines. Higher LINC00483 levels closely correlated to shorter survival times, advanced TNM stage, larger tumor size and positive lymph node metastasis. Cell proliferation, migration and invasion were suppressed after LINC00483 knockdown. LINC00483 mainly localized in the cytoplasm, where it acted as a sponge of miR-204-3p. ETS1 was validated as a downstream target of miR-204-3p and is thus regulated by LINC00483.

**Conclusion:**

This study demonstrated that LINC00483 facilitates the proliferation, migration and invasion of LUAD cells by acting as a sponge for miR-204-3p, which in turn regulates ETS1.

## Introduction

Lung cancer accounts for about one-quarter of cancer mortality, owing to its high invasiveness and rapid metastasis [1, 2]. Non-small cell lung cancers (NCSLCs) account for more than 80% of all lung cancer cases. More than a half of NCSLCs are lung adenocarcinoma (LUAD), also known as pulmonary adenocarcinoma. The survival rate of LUAD patients remains unsatisfactory despite the developments made in early diagnosis and treatment [3]. Exploring the molecular mechanisms underlying LUAD progression is of great importance to improving patient survival rates.

Non-coding RNAs are a class of RNAs that do not translate into proteins. The class includes long noncoding RNAs (lncRNAs), which are transcripts larger than 200 bp [4]. Increasing evidence has shown that mutations and aberrant expression of lncRNAs play a crucial role in cancers including LUAD [5, 6].

Poor prognosis for colorectal cancer patients is associated with aberrant expression of the lncRNA LINC00483 [7], which is located 20 kbp upstream of the SPAG9 gene. Although a TCGA database analysis and expression level determination from clinical samples show that LINC00483 is upregulated in LUAD tissues, its role in the progression of this malignancy has not been elaborated.

MicroRNAs are another class of non-coding RNAs. They are about 22 nucleotides in length. MicroRNAs are implicated in various physiological processes, including cancer development at the post-transcriptional level [8]. MiR-204-3p is significantly downregulated in hepatocellular carcinoma (HCC), while its upregulation can induce HCC cell apoptosis [9]. Bioinformatics predictions indicate that miR-204-3p is a downstream target of LINC00483, but there have been no studies on a potential role in LUAD progression.

A predicted target of miR-204-3p is ETS1. It belongs to ETS family of transcription factors, each of which contains a unique DNA-binding domain [10]. High expression of ETS1 was found to correlate to poor clinical outcomes, such as increased distant metastasis and higher tumor grading in lung cancer [11]. Elevated ETS1 is also associated with upregulation of urokinase-type plasminogen activator, an critical invasion-promoting factor [12]. As with the roles of LINC00483 and miR-204-3p, any association between miR-204-3p and ETS1 in LUAD remains elusive.

To validate the relationships between LINC00483, miR-204-3p and ETS1 specifically for the context of LUAD, we examined the expression levels of the two RNAs in LUAD and paracancerous tissues. We also explored the effects of LINC00483 and miR-204-3p on the proliferation, migration and invasion of LUAD cells. Finally, we validated the predicted correlation.

## Materials and methods

### Clinical samples and ethics statement

Patients (*n* = 60) gave written informed consent for all collection of LUAD and paracancerous tissue samples. All experiments involving clinical samples were performed following the ethical guidelines of the Declaration of Helsinki and were approved by the Ethics Committee of the Second Affiliated Hospital of Guangxi Medical University.

### Tumor xenograft

Healthy 4- to 6-week old BALB/c nude mice were purchased from Uban. A549 cells transfected with si-NC or si-LINC00483 were cultured to the logarithmic phase, then 5 × 10^6^ cells/mouse were subcutaneously injected into the mice (*n* = 6). The mice were killed 27 days after the inoculation. The tumor tissues were measured and fixed for subsequent quantitative real-time PCR and western blotting. All the animal investigations were performed following the guidelines for the care and use of laboratory animals and approved by the Ethics Committee of the Second Affiliated Hospital of Guangxi Medical University.

### Cell culture and transfection

Four LUAD cell lines (A549, SPC-A1, PC-9 and H1975) and the pulmonary epithelial cell line BEAS-2B were purchased from the cell bank of the Chinese Academy of Sciences. All cell lines were cultured in RPMI-1640 culture medium with 10% fetal bovine serum (FBS) in a humidified incubator with 5% CO_2_ at 37 °C. LINC00483 was inserted into pcDNA3.1 plasmid between Hind III and Xho I to overexpress LINC00483. A549 and PC-9 cell lines were transfected with siRNAs or plasmids using Lipofectamine 2000 (Invitrogen). SiRNAs, miRNA mimics and miRNA inhibitors were directly synthesized by Sangon.

### Quantitative real-time PCR

Total RNA from cells and tissues was extracted with Trizol reagent (Invitrogen) Super M-MLV reverse transcriptase (Beyotime) was used for reverse transcription. SYBR Green (Sigma) was used to perform the quantitative real-time PCR. PCR data were analyzed using the 2^-△△CT^ method. GAPDH and RNU6B (U6) were used as internal references for the detection of RNA levels. The real-time primers were:

**Table:** 

LINC00483	Forward: 5′-GCTGAACCGGAACAGGACAT-3′
	Reverse: 5′-CCAGTTCACAGCAACTCACG-3′
miR-204-3p	Forward: 5′-TGTTGCAGTGAGGGCAAGAA-3′
	Reverse: 5′-GACCCTGGTTGCTTCAAGGA-3′
GAPDH	Forward: 5′-AACTTTGGTATCGTGGAAGGAC-3′
	Reverse: 5′-GCAGGGATGATGTTCTGGAG-3′
U6	Forward: 5′-CTCGCTTCGGCAGCACA-3′
	Reverse: 5′-AACGCTTCACGAATTTGCGT-3′

### Western blotting assay

Cells and tissues were lysed with RIPA lysis buffer (Beyotime). After quantification with a BCA kit, total proteins were separated using sodium dodecyl sulfate polyacrylamide gel electrophoresis (SDS-PAGE). The proteins were then transferred onto a PVDF membrane (Millipore) and sealed with 5% skim milk. The PVDF membranes were then incubated with primary antibodies overnight at 4 °C, followed by incubation with HRP-conjugated goat anti-rabbit IgG and HRP-conjugated goat anti-mouse IgG (1:5000, Proteintech) for 60 min at 37 °C. Primary antibodies: rabbit anti-ETS1 antibody (1:1000, Abcam), rabbit anti-Snail antibody (1:1000, Abcam), rabbit anti-E-cadherin antibody (1:500, Proteintech), rabbit anti-N-cadherin (1:1000, Proteintech) and mouse anti-GAPDH antibody (1:3000, Proteintech).

### Cell counting Kit-8 assay

Cell proliferation was assessed using the Cell Counting Kit-8 (CCK8) assay. A549 and PC-9 cells were seeded into 96-well plates (2 × 10^3^ cells/well) and transfected with siRNAs, followed by incubation with 10 μl of CCK-8 solution (Glpbio) for 2 h on days 1, 2, 3 and 4 after transfection. The optical density (OD) value at 450 nm was recorded.

### Colony formation assay

A549 and PC-9 cells at the logarithmic growth phase were seeded into 6-well plates (1 × 10^3^ cells/well) and transfected with siRNAs, followed by 14 days’ culture with RPMI-1640 medium until cell colonies were visible. Cells were then fixed with methanol for 15 min and stained with Giemsa for 20 min. The number of clusters with more than 50 cells was counted.

### Transwell assays

A549 and PC-9 cells (2 × 10^4^ cells/ml) were seeded on the upper chamber of a Corning transwell setup pre-coated with Matrigel (for the cell invasion assay) or nothing (for the cell migration assay). Serum-free culture medium was added into upper chamber and culture medium containing 10% FBS was used as an attractant in the lower chamber. Cells were immobilized with paraformaldehyde and stained with crystal violet. The migrating and invading cells were observed and counted under a microscope.

### Fluorescence in situ hybridization (FISH)

FISH was performed to investigate the localization of LINC00483 in the cells. In brief, A549 and PC-9 cells in the logarithmic growth phase were seeded in a 6-well plate. Cells were then cultured for 24 h and fixed with 4% polyoxymethylene, followed by incubation for 1 h with prehybridization solution at 42 °C. The cells were then hybridized with 250 μl hybridization solution (cy3-labelled LINC00483, RiboBio) overnight at 42 °C. After that, cells were stained with DAPI, sealed with anti-fluorescence quenching agent, and observed under a fluorescence microscope.

### RNA immunoprecipitation

The RNA immunoprecipitation assay was used to detect the binding of LINC00483 and miR-204-3p to the Argonaute 2 (Ago2) protein. A549 and PC-9 cells were lysed with RIPA buffer (Bioteke). Part of the protein supernatant was used as input and the rest was used for subsequent immunoprecipitation. The supernatant was incubated with anti-Ago2-coated agarose beads (MBL) overnight at 4 °C. The enriched RNA–protein complex was analyzed with real-time PCR and western blotting with anti-Ago2 antibody (1:2000, Abcam).

### Dual-luciferase assay

The luciferase reporter assay was performed to verify the relationships between LINC00483 and miR-204-3p and between miR-204-3p and ETS1. Mutant-type LINC00483 and ETS1 fragments absent of the miR-204-3p-binding site were obtained using overlapping PCR and cloned into the pUM-T vector (Bioteke). Then wild- and mutant-type fragments were amplified and inserted into the pmiRGLO plasmid between Sac I and Xho I. A549 and PC-9 cells were co-transfected with the constructed pmiRGLO plasmids and miR-204-3p or miR-NC for 48 h. A commercial luciferase assay kit (KeyGEN) was used to determine the luciferase activity (firefly luciferase activity/renilla luciferase activity).

### Statistical analysis

GraphPad Prism 7 was used for data analysis. Results were displayed as means ± SD. Mean values between two groups were compared with Student’s t-test. The remaining data were analyzed using one-way ANOVA. The correlation between the clinicopathological characteristics of patients with LUAD and the LINC00483 expression level was analyzed with Pearson χ^2^ test. All experiments were repeated more than three times and *p* < 0.05 was regarded as statistically significant.

## Results

### The expression of LINC00483 is elevated in LUAD tissues and cell lines

**Fig. 1:**
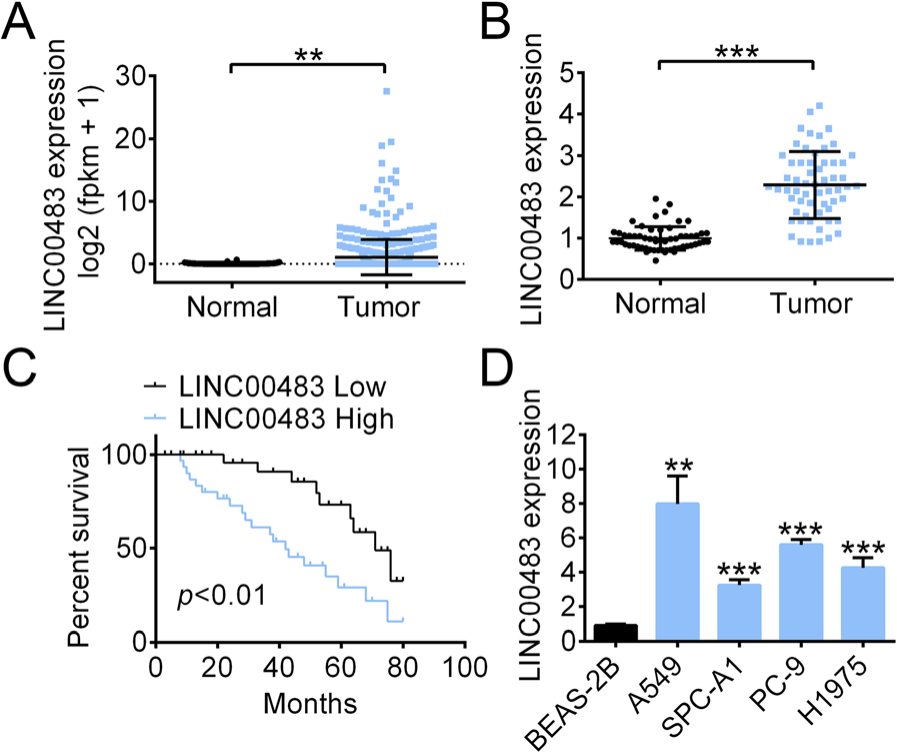
LINC00483 expression was upregulated in LUAD tissues and cells. **a** Analysis of LINC00483 expression in LUAD tissues and normal tissues based on the cancer genome atlas database. **b** The RNA level of LINC00483 in LUAD tissues and normal tissues was measured using quantitative real-time PCR (*n* = 60). **c** Analysis of correlation between the survival time of LUAD patients and LINC00483 expression. **d** The RNA level of LINC00483 in cell lines was measured using real-time PCR. LUAD: lung adenocarcinoma, **p < 0.01; ***p < 0.001

According to the analysis in the Cancer Genome Atlas database, LINC00483 had higher expression in LUAD tissues than in adjacent non-tumor tissues (Fig. 1a). We also determined the expression of LINC00483 in LUAD tissues and non-tumor tissues (*n* = 60 in each group), finding that it was upregulated in the tumor tissues (Fig. 1b).

Patients with higher LINC00483 levels had shorter overall survival times as compared to those with low expression (Fig. 1c). We also found that the expression level of LINC00483 in LUAD cell lines was significantly higher than that in the pulmonary epithelial cell line BEAS-2B. In particular, A549 and PC-9 cell lines displayed higher LINC00483 levels than BESA-2B and H1975 cell lines (Fig. 1d).

### LINC00483 expression is correlated with poor prognosis for LUAD patients

The clinicopathological characteristics of 60 independent LUAD patients were investigated and a correlation analysis was performed. It is noteworthy that advanced TNM stage (*p* = 0.028), larger tumor size (*p* = 0.006) and positive lymph node metastasis (*p* = 0.008) displayed positive correlations with higher LINC00483 expression (Table 1). However, no significant relationship was observed between LINC00483 expression and age (*p* = 0.438), gender (*p* = 0.426), and smoking history (*p* = 0.796). These results suggested that higher LINC00483 expression is associated with poor prognosis for LUAD patients.

**Table 1:** The correlation between LINC00483 RNA level and clinicopathological characteristics as analyzed using chi-square tests

Characteristics	N (%)	LINC00483		*c*^*2*^ value	*p* value
		Low	High		
Age (years)				0.601	0.438
> 65	31 (51.67)	14	17		
≤ 65	29 (48.33)	16	13		
Gender				0.635	0.426
Male	23 (38.33)	10	13		
Female	37 (61.67)	20	17		
TNM stage				4.800	0.028
I + II	22 (36.67)	6	14		
IIIa	38 (63.33)	24	16		
Tumor size				7.500	0.006
≤ 5	20 (33.33)	5	15		
> 5	40 (66.67)	25	15		
Lymph node metastasis				6.944	0.008
Negative	24 (40.00)	7	17		
Positive	36 (60.00)	23	13		
Smoking history				0.067	0.796
Smokers	31 (51.67)	15	16		
Never smokers	29 (48.33)	15	14		

### LINC00483 knockdown suppresses the proliferation, migration and invasion of LUAD cells

**Fig. 2:**
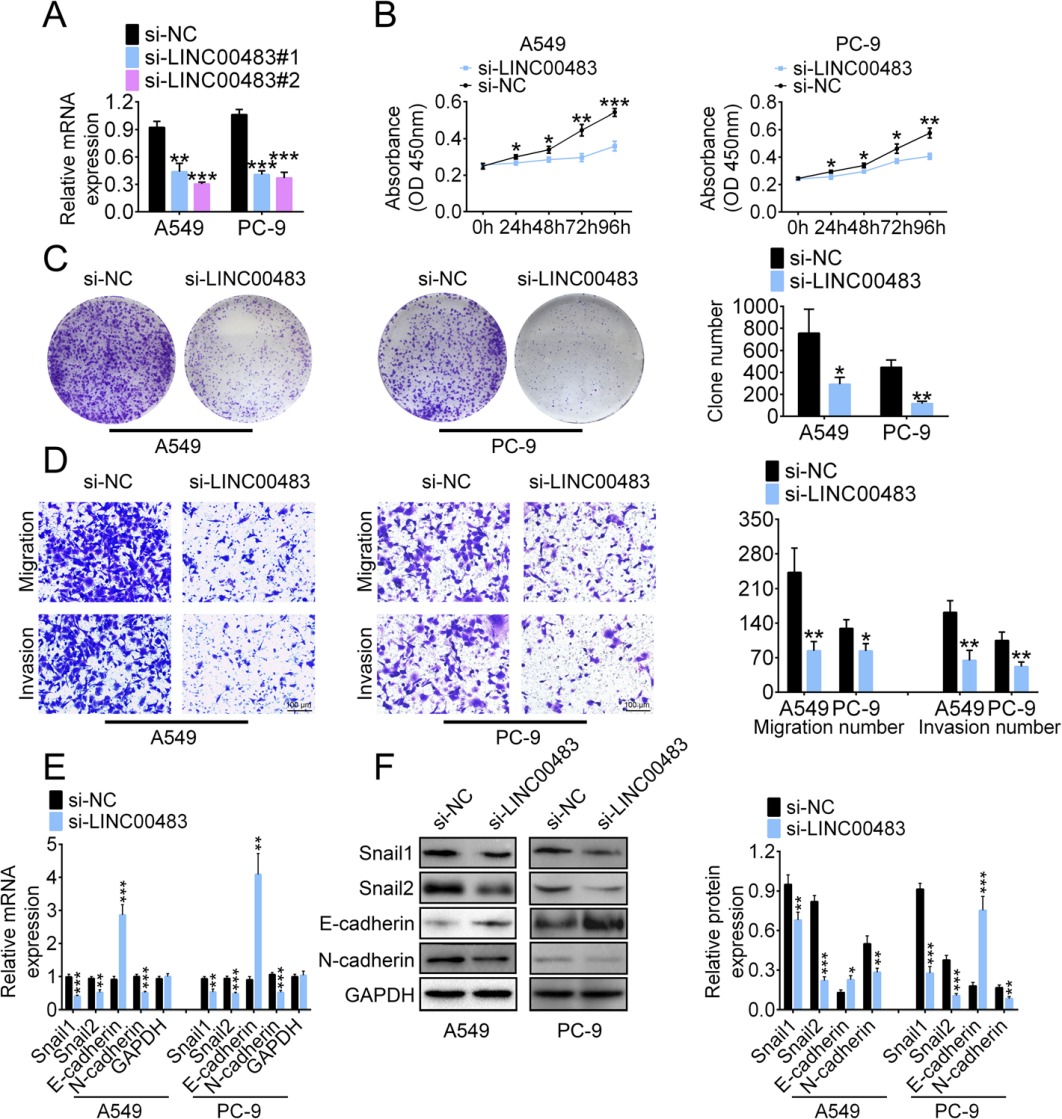
LINC00483 knockdown suppresses the proliferation, migration and invasion of LUAD cells. **a** Knockdown efficiency of LINC00483 was evaluated using real-time PCR. **b** The proliferation of A549 and PC-9 cells was assessed using the Cell Counting Kit-8 assay. **c** The colony formation assay was also performed to evaluate cell proliferation. **d** Migration and invasion of A549 and PC-9 cells were evaluated using a transwell assay. **e** and **f** The relative RNA and protein levels of Snail, Snail2, E-cadherin and N-cadherin were measured using real-time PCR € and western blotting (**f**), *p < 0.05; **p < 0.01; ***p < 0.001

To explore the role of LINC00483 in the progression of LUAD, A549 and PC-9 cells were transfected with two LINC00483 siRNAs (si-LINC00483 #1 and si-LINC00483 #2). This significantly inhibited LINC00483, with si-LINC00483 #2 being more efficient and thus being used for our subsequent investigations (Fig. 2a). The proliferation of A549 and PC-9 cells was significantly inhibited after si-LINC00483 transfection compared to the proliferation of cells transfected with si-NC, and the impact was time dependent (Fig. 2b).

Cell proliferation was also evaluated using the colony formation assay. The clone number of A549 and PC-9 cells notably decreased after LINC00483 knockdown (Fig. 2c). In the transwell assay, LINC00483 knockdown markedly inhibited the migration and invasion of A549 and PC-9 cells (Fig. 2d). Furthermore, the mRNA levels of the epithelial–mesenchymal transition (EMT) markers Snail1, Snail2 and N-cadherin significantly decreased after si-LINC00483 transfection, but the mRNA level of E-cadherin increased (Fig. 2e). The western bloting assay showed consistent results (Fig. 2f). These results indicate that LINC00483 knockdown could inhibit the proliferation, migration and invasion of LUAD cells in vitro.

### LINC00483 acts as a sponge of miR-204-3p

**Fig. 3:**
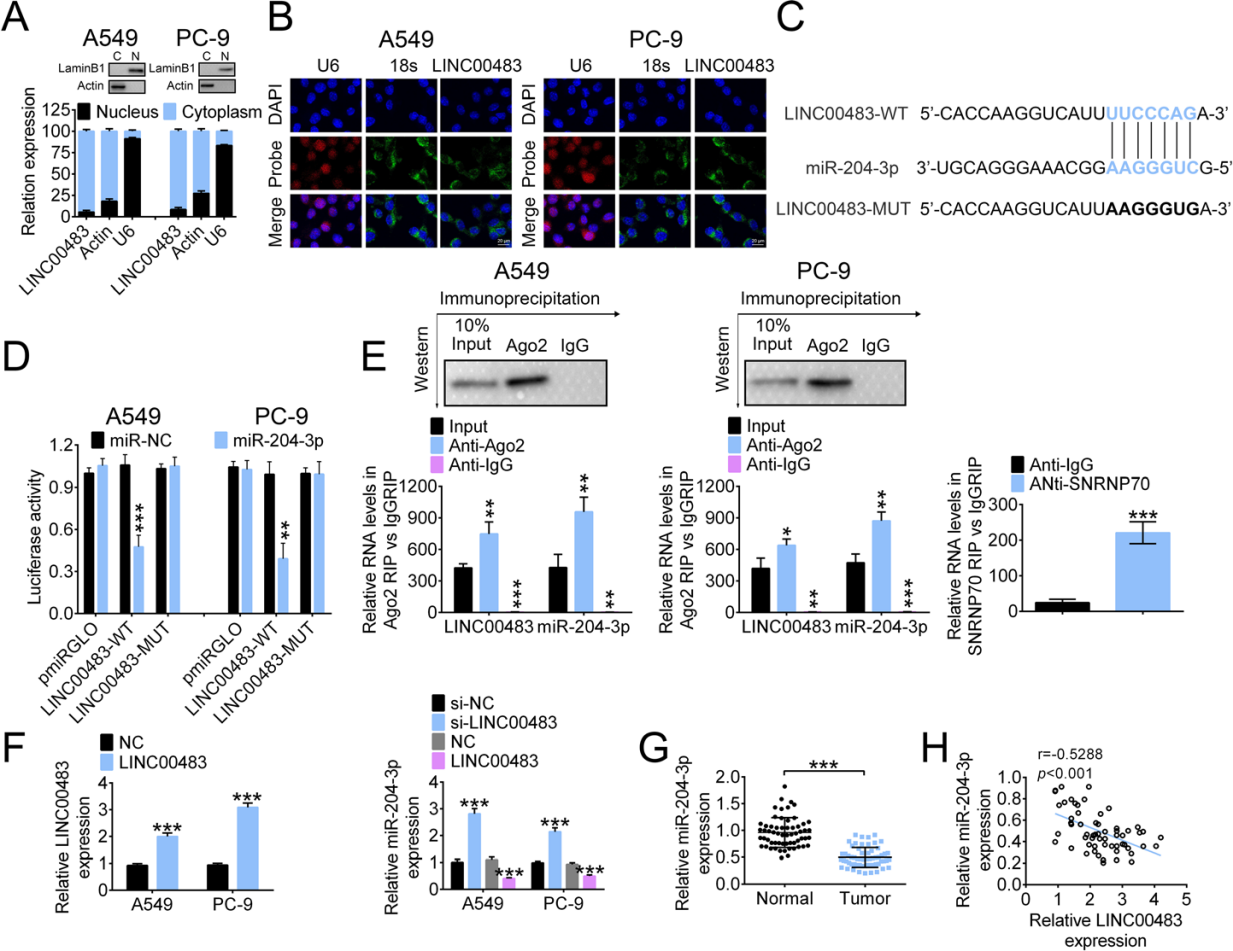
LINC00483 acts as a sponge of miR-204-3p. **a** The expression of LINC00483 in the cytoplasm and nucleus of A549 and PC-9 cells was measured using real-time PCR. **b** Fluorescence in situ hybridization assay was performed to determine the subcellular localization of LINC00483. **c** and **d** The correlation between LINC00483 (**c**) and miR-204-3p (**d**) was validated using the dual-luciferase assay. **e** LINC00483 and miR-204-3p were enriched via RNA immunoprecipitation with Ago2 antibody. SNRNP70 was used as a control. **f** The RNA levels of LINC00483 and miR-204-3p after LINC00483 overexpression were measured using real-time PCR. **g** and **h** The expression of miR-204-3p in tumor and normal tissues was measured using real-time PCR, and a negative correlation between LINC00483 level and miR-204-3p expression was observed, **p < 0.01; ***p < 0.001

Our real-time PCR and FISH results show that LINC00483 is mainly expressed in the cytoplasm (Fig. 3a and b). The binding sites between miR-204-3p and LINC00483 were predicted using miRDB (http://www.mirdb.org/; Fig. 3c). The luciferase activity of cells co-transfected with wild-type LINC00483 (LINC00483-WT) and miR-204-3p was significantly lower than that for cells co-transfected with LINC00483-WT and miR-NC. By contrast, no difference in luciferase activity was detected between cells co-transfected with mutant LINC00483 (LINC00483-MUT) and miR-NC and cells co-transfected with LINC00483-MUT and miR-204-3p (Fig. 3d).

In an RNA immunoprecipitation assay with Ago2 antibody and SNRNP70 as a control, Ago2 protein levels were successfully pulled down, and LINC00483 and miR-204-3p were significantly enriched compared to the control (Fig. 3e). We further transfected pcDNA3-LINC00483 into A549 and PC-9 cells, and the expression of miR-204-3p was significantly decreased upon LINC00483 overexpression. The expression of miR-204-3p was notably elevated after LINC00483 knockdown (Fig. 3f). In addition, the RNA level of miR-204-3p in LUAD tissues was markedly lower than in non-tumor tissues (Fig. 3g). The miR-204-3p level negatively correlated to LINC00483 expression (Fig. 3h).

### ETS1 is a downstream target of miR-204-3p

**Fig. 4:**
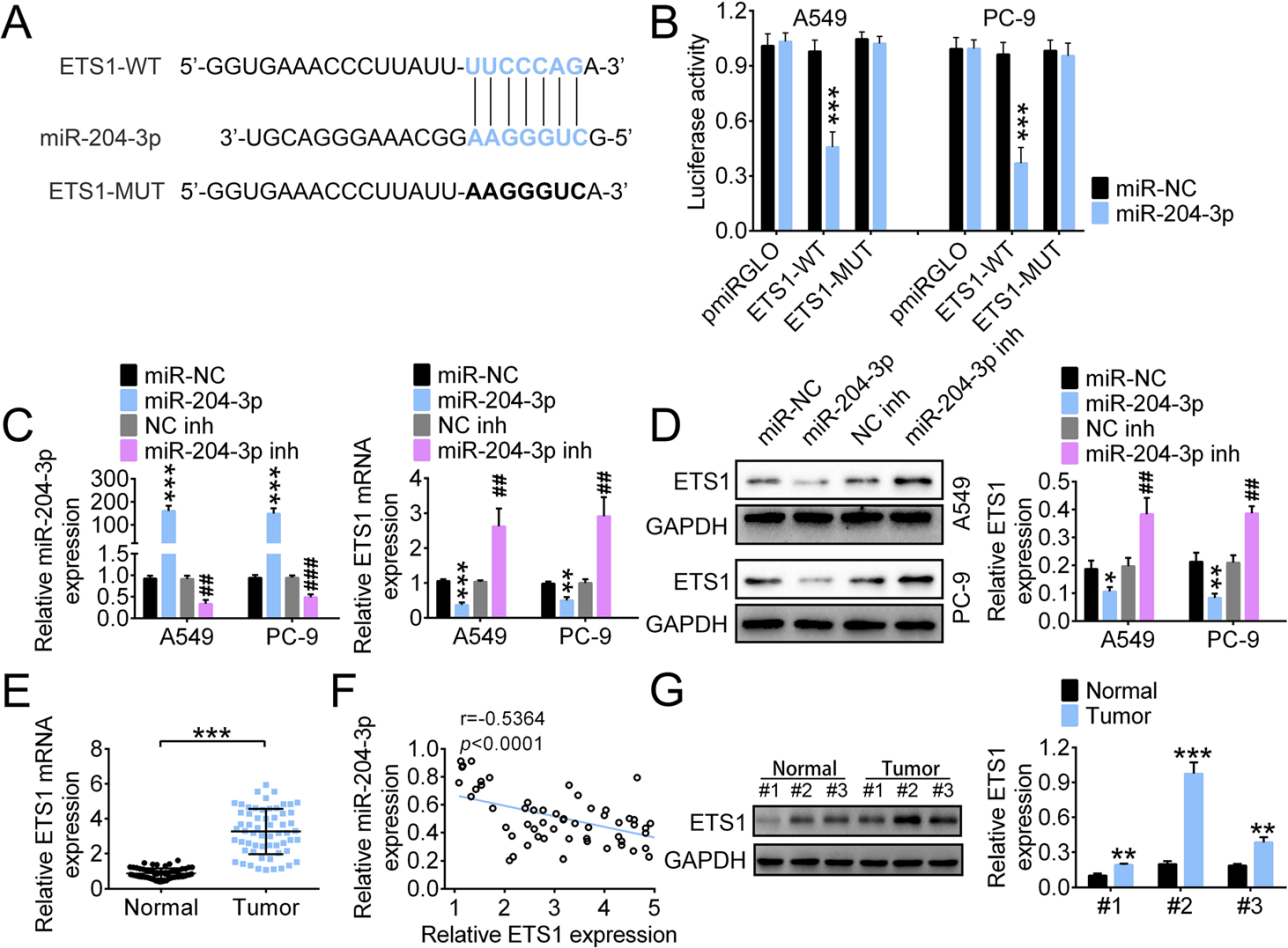
ETS1 is a target gene of miR-204-3p. **a** and **b** The correlation between miR-204-3p (**a**) and ETS1 (**b**) was validated using the dual-luciferase assay. **c** The mRNA levels of miR-204-3p and ETS1 in A549 and PC-9 cells transfected with miR-204-3p mimics or miR-204-3p inhibitor were measured using real-time PCR. **d** The protein level of ETS1 in A549 and PC-9 cells transfected with miR-204-3p mimics or miR-204-3p inhibitor was determined via western blotting. **e** and **f** The expression of ETS1 in tumor and normal tissues was measured using real-time PCR and a negative correlation between miR-204-3p level and ETS1 expression was observed. **g** The protein level of ETS1 in tumor and normal tissues was detected using western blotting. *p < 0.05; **p < 0.01; ##*p* < 0.01; ***p < 0.001

Targetscan (http://www.targetscan.org/vert_71/) predictions indicate that ETS1 is a candidate downstream target of miR-204-3p. The dual-luciferase assay was performed to validate the correlation between miR-204-3p and ETS1. The binding sites between miR-204-3p and ETS1 are shown in Fig. 4a.

A549 and PC-9 cells co-transfected with ETS1-WT and miR-204-3p showed significantly decreased luciferase activity compared to cells co-transfected with ETS1-WT and miR-NC (Fig. 4b). The level of miR-204-3p was markedly elevated in A549 and PC-9 cells transfected with miR-204-3p mimics, but significantly decreased by the miR-204-3p inhibitor. Indeed, the ETS1 RNA level in A549 and PC-9 cells was notably downregulated after transfection of miR-204-3p mimics, but was upregulated by the miR-204-3p inhibitor (Fig. 4c). Western blotting showed consistent results (Fig. 4d). We further found that a higher ETS1 RNA level was observed in LUAD tissues than in non-tumor tissues (Fig. 4e). The level of miR-204-3p negatively correlated to that of ETS1 (Fig. 4f). We also determined the protein level of ETS1 in tumor and normal tissues (*n* = 3 in each group). Higher ETS1 expression was observed in LUAD tissues than in normal tissues (Fig. 4g).

### LINC00483 promotes the proliferation, migration and invasion of LUAD cells by regulating miR-204-3p

**Fig. 5:**
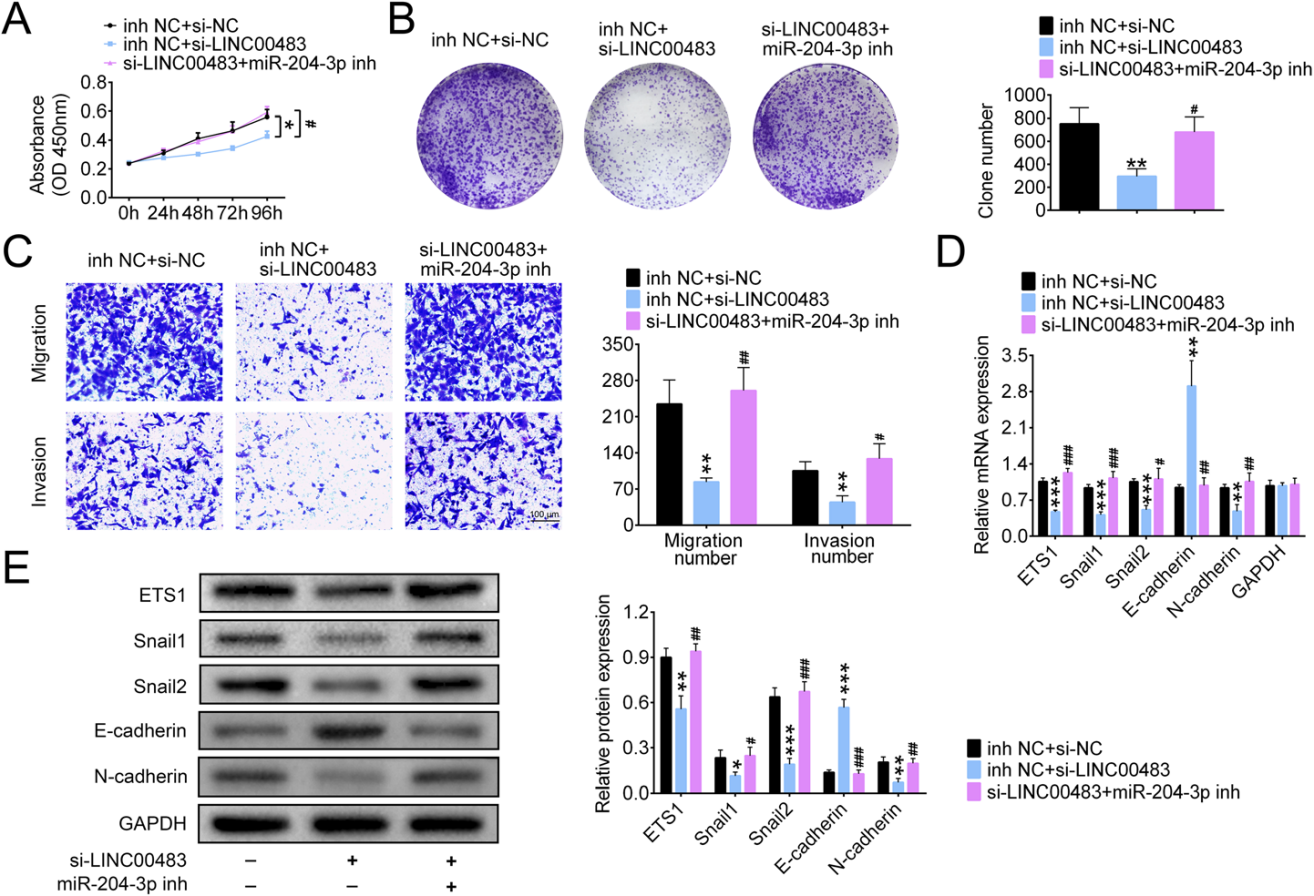
LINC00483 promotes the proliferation, migration and invasion of LUAD cells by inhibiting miR-204-3p. **a** and **b** The proliferation of A549 cells was assessed using the Cell Counting Kit-8 (**a**) and colony formation (**b**) assays. **c** The migration and invasion of A549 cells after LINC00483 silencing and miR-204-3p inhibition were assessed using a transwell assay. **d** and **e** The mRNA and protein levels of ETS1, Snail1, Snail2, E-cadherin and N-cadherin in A549 cells were measured using real-time PCR and western blotting, *p < 0.05; *^#^*p* < 0.05; **p < 0.01; ^##^*p* < 0.01; ***p < 0.001; ^###^*p* < 0.001

The role of miR-204-3p in the proliferation, migration and invasion of A549 cells was investigated using the miR-204-3p inhibitor. The proliferation of A549 cells was significantly suppressed after LINC00483 knockdown, but miR-204-3p inhibition largely alleviated this suppression (Fig. 5a). This result was further validated with the colony formation assay, evidenced by the increase in cell clone number after transfection with the miR-204-3p inhibitor (Fig. 5b). The migration and invasion of A549 cells were also inhibited by LINC00483 knockdown, and this effect was abrogated after transfection with the miR-204-3p inhibitor (Fig. 5c). In addition, the expression levels of ETS1, Snail1, snail2 and N-cadherin were downregulated after LINC00483 knockdown, but miR-204-3p inhibition reversed this effect. Expression of E-cadherin was upregulated by LINC00483 knockdown, and this elevation was abolished after transfection with the miR-204-3p inhibitor (Fig. 5d and e).

### LINC00483 knockdown inhibited tumor growth and downregulated ETS1 expression

**Fig. 6:**
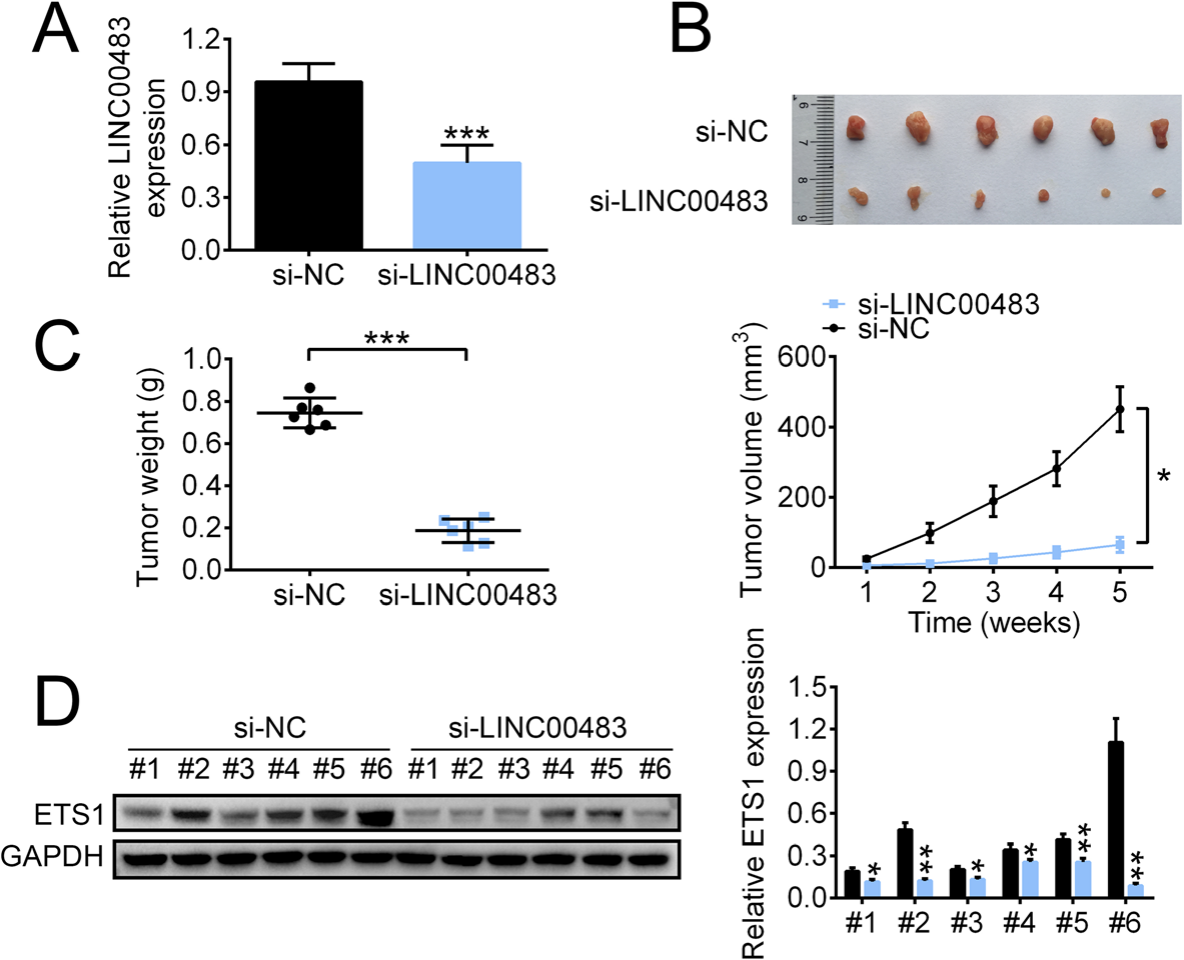
LINC00483 silencing inhibited tumor growth and decreased ETS1 expression. **a** The RNA level of LINC00483 after LINC00483 silencing was measured using real-time PCR. **b** and **c** Tumor weight (**b**) and volume (**c**) were measured after LINC00483 silencing. **d** The protein level of ETS1 in tumors was determined using western blotting, *p < 0.05; ***p < 0.001

Finally, we explored the effect of LINC00483 on tumor growth in vivo. A549 cells transfected with si-NC or si-LINC00483 were subcutaneously injected into mice, and a significant decrease in LINC00483 level was observed in the si-LINC00483 group (Fig. 6a). Tumors derived from A549 cells transfected with si-LINC00483 (si-LINC00483 tumors) showed obviously lower tumor weight and smaller tumor volume than tumors derived from si-NC-transfected A549 cells (si-NC tumors; Fig. 6b and c). In addition, the mRNA and protein levels of ETS1 were lower in tumors derived from si-LINC00483 than in those derived from si-NC (Fig. 6d).

## Discussion

Although tremendous progress has been made in the understanding of mechanisms implicated in lung tumorigenesis, the five-year relative survival rate of lung cancer patients is still unsatisfactory, especially in patients with distant metastasis (only about 5%) [13]. The major obstacle for lung cancer treatment is late-stage diagnosis, which leaves very limited therapeutic options with little success rate [14]. Reliable new prognostic biomarkers for lung cancer would have great significance.

LncRNAs are a class of versatile RNAs involved in tumor initiation, progression and metastasis at the epigenetic, transcriptional, and post-transcriptional levels [15, 16]. Increasing evidence shows that lncRNAs are aberrantly expressed in lung cancers and closely related to the clinical outcome in lung cancer patients. For instance, MALAT1, one of the identified cancer-related lncRNAs, was reported to be closely associated with distant metastasis in NSCLC patients [17]. LncRNA regulator of reprogramming (linc-ROR) had a higher expression in NCSLC tissues than adjacent non-tumor tissues, and this elevated linc-ROR expression positively correlated to advanced TNM stage and lower five-year overall survival [18]. Also, lncRNA bladder cancer associated transcript 1 (BLACAT1) was upregulated in both NCSLC tissues and cells, with its elevated expression facilitating the proliferation and invasion of NCSLC cells [19].

Although many lncRNAs have been discovered, their exact function in cancers and the underlying mechanisms still require deeper study. Here, we investigated the role that LINC00483, a functional lncRNA discovered in 2017, plays in the most common lung cancer: lung adenocarcinoma. LINC00483 was found to be upregulated in LUAD tissues and cells. This pattern was also reported for colorectal and gastric cancer cells in previous studies [7, 20]. Elevated LINC00483 expression positively correlated to shorter survival time, advanced TNM stage, larger tumors and positive lymph node metastasis. An integrated bioinformatics analysis showed that LINC00483 has prognostic power in endometrial carcinoma [21].

Malignant proliferation of cancer cells plays a critical role in the development and progression of cancers [22]. LINC00483 knockdown led to an obvious inhibition of LUAD cell proliferation in this study. It could also suppress tumor growth in vivo.

The consequences of abnormal cell migration include tumor formation, while invasive cancer cells can permeate into nearby tissues and further trigger distant metastasis [23, 24]. Approximately 90% of LUAD death results from distant metastasis of cancer cells to other organs.

EMT is a crucial process by which epithelial cells acquire the invasiveness of mesenchymal cells, which facilitates cancer invasiveness and metastasis [25, 26]. In our study, LINC00483 knockdown suppressed the migration and invasion of LUAD cells and this was accompanied with changes in the expression level of EMT-related markers: Snails and N-cadherin were downregulated while E-cadherin expression was elevated. Our findings were highly consistent with those of a previous study that showed that LINC00483 silencing inhibited EMT by interacting with HOXA10 in LUAD [27]. These results indicate that LINC00483 promotes the proliferation and invasion of LUAD cells and may further facilitate cancer metastasis.

LncRNAs can “talk to” microRNAs according to the “competitive endogenous RNA (ceRNA)” hypothesis. In this study, we discovered that the microRNA miR-204-3p directly interacts with LINC00483. LINC00483 is mainly expressed in cytoplasm where it acts as a sponge of miR-204-3p, as validated using the luciferase reporter assay. Furthermore, RNA immunoprecipitation with Ago2 revealed that LINC00483 and miR-204-3p are highly enriched in LUAD cells. The expression of LINC00483 negatively correlated to that of miR-204-3p in both LUAD tissues and cells. MiR-204-3p was downregulated in tumor tissues, and overexpression of miR-204-3p inhibited proliferation, migration and invasion while promoting apoptosis in several cancers [9, 28, 29]. In particular, the inhibition of proliferation and invasion caused by LINC00483 silencing was abolished after miR-204-3p inhibition. This was in accordance with the anti-tumor effect reported in previous studies [9, 30].

All these results suggest that LINC00483 exerts its tumor-promoting function by regulating miR-204-3p. This new regulatory axis may provide novel therapeutic target for LUAD treatment.

In addition, we validated that ETS1 is a downstream target gene of miR-204-3p and that ETS1 expression positively correlates to LINC00483 level. ETS1 is upregulated in cancer cells and is linked to poor clinical outcome in patients, so it may serve as a diagnostic marker [11, 31, 32]. ETS1 also facilitated the acquisition of invasiveness, drug resistance and neo-angiogenesis in cancer cells [11].

Our results showed that LINC00483 promoted LUAD progression by sponging miR-204-3p and further restoring ETS1. This gives further information about this new regulatory axis for LUAD development.

Although solid work has been performed to investigate the role of LINC00483 in lung adenocarcinoma, our study still has limitations. We did not investigate the impact of LINC00483 on tumor metastasis in the mouse model, and the promotion of EMT mediated by LINC00483 also needs more experimental proof.

## Conclusion

Our study demonstrates that LINC00483 promotes the progression of lung adenocarcinoma by sponging miR-204-3p. Our results hint that LINC00483 might serve as a diagnostic marker and therapeutic target for lung adenocarcinoma.

### Ethics approval and consent to participate

The animal use protocol was reviewed and approved by the Animal Ethical and Welfare Committee of The Second Affiliated Hospital of Guangxi Medical University, China.

## Data Availability

All data generated or analyzed during this study are included in this published article.

## Abbreviations

HCC: Hepatocellular carcinoma
LncRNAs: Long noncoding RNAs
LUAD: Lung adenocarcinoma
NCSLCs: Non-small cell lung cancers
